# Supplementary material for: Increasing CB2 Receptor Activity after Early Life Stress Prevents Depressive Behavior in Female Rats
Source: Biomolecules. 2024 Apr 10;14(4):464. doi: 10.3390/biom14040464 (PMC11047932; doi:10.3390/biom14040464)
Supplement: Supplementary file 1 [file biomolecules-14-00464-s001.zip › biomolecules-2842664-supplementary.pdf]

Supplementary information:

Figure S1. Western immunoblot of MARCH7 with VCP control.

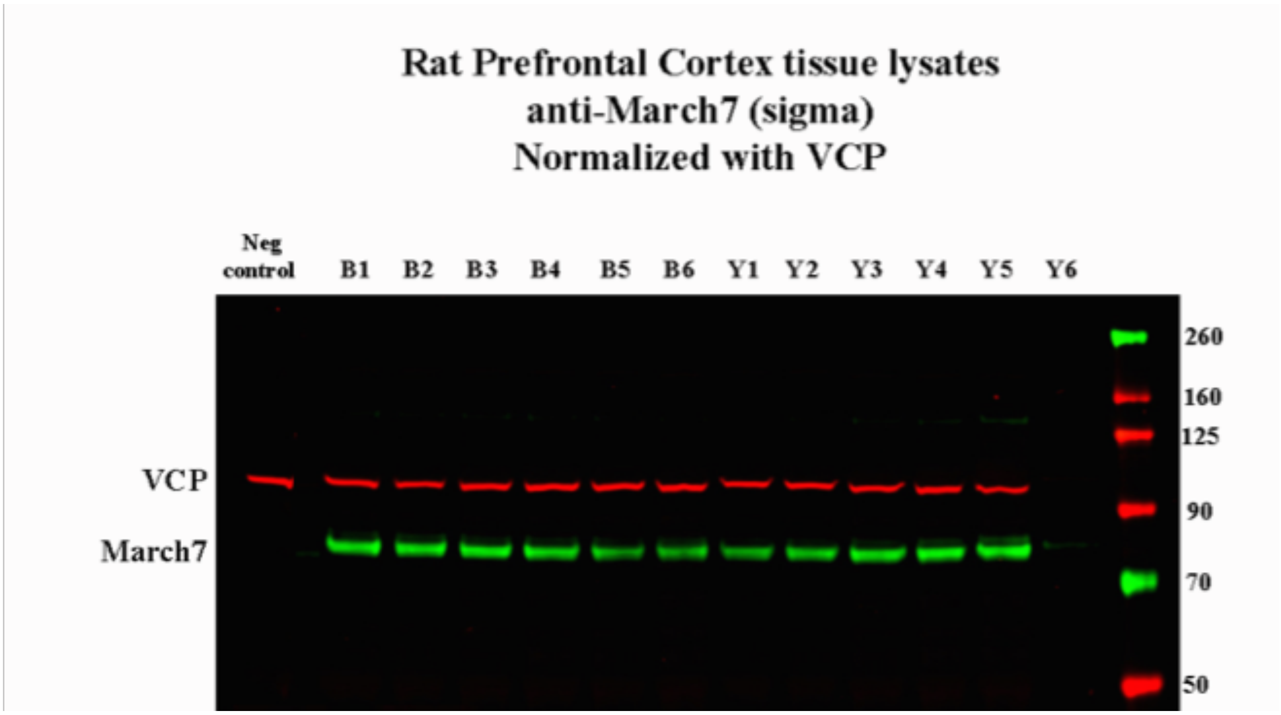

Controls: B1-B6; Maternal Separation Y1-Y6. Marker
